# Examination of Psychotropic Medication Use Following Outpatient Behavioral Assessment and Treatment

**DOI:** 10.3390/brainsci15050513

**Published:** 2025-05-17

**Authors:** Maria G. Valdovinos, Melissa Trites, Janelle Ausenhus

**Affiliations:** 1 Department of Psychology and Neuroscience, College of Arts and Scienced, Drake University, Des Moines, IA 50311, USA; 2 Heartland Area Education Agency, Johnston, IA 50131, USA; 3 Organizational Management and Communication Program, College of Applied Studies, Florida State University, Panama City, FL 32306, USA; jp12m@fsu.edu

**Keywords:** psychotropic medication, challenging behavior, functional assessment

## Abstract

**Background/Objectives:** Psychotropic medications are often prescribed to treat challenging behavior in children with neurodevelopmental disorders. This study examined patterns of psychotropic medication use following outpatient behavioral assessment and treatment in children ages 2–16 years. **Methods:** Medication use at the time of behavioral assessments, six months after the assessment, and a later follow-up time point (mean 25 months following the six-month time point, range 1 month to 41 months) were evaluated via a chart review. Alterations in psychotropic medication use were grouped into eight categories according to the type of medication change experienced. Care providers also completed a social validity survey rating their satisfaction with the assessment and interventions developed for their child. **Results:** This retrospective study revealed that children in this sample were more likely to experience starting a new medication and increases in the dose of psychotropic medication as time passed. Children were also less likely to remain on the same regimen of psychotropic medication as when they were first seen in the clinic. Additionally, although caregivers generally rated their experiences and outcomes with the behavioral clinic as favorable, additions and increases to psychotropic medication regimens still occurred. **Conclusions:** These findings are consistent with other reports of continued and increased prescribing of psychotropic medication across time in children with neurodevelopmental disorders, however, the results must be interpreted with caution given the small sample size which limits generalizability of these findings. Additionally, the lack of follow-up with the patients in this sample made it difficult to correlate changes in challenging behavior with psychotropic medication prescribing.

## 1. Introduction

Challenging behavior is often observed in children with neurodevelopmental disorders although the prevalence of these behaviors varies greatly. For example, a meta-analysis [1] of the existing literature on challenging behavior in those with intellectual disabilities found that between the ages of 2 and 14, the percentage of children who engaged in aggression ranged from 0% to 35% and in adolescents between the ages of 15 to 20 years of age, the range was between 11.62% to 33%. For SIB, 0% to 30% of children between the same ages were reported to engage in SIB and for adolescents, the reported range was 7.7% to 22.2%.

Behavior analysts have a long history of demonstrating how the application of behavior analytic principles and techniques are effective interventions for addressing these challenging behaviors. Although some have criticized the lack of clinical trial evaluation regarding the effectiveness of behavior analytic interventions for addressing challenging behavior, meta-analyses of the existing literature and consecutive controlled case series designs have demonstrated evidence to support the effectiveness of behavioral interventions in reducing challenging behavior [2,3].

An additional benefit of behavioral technology is that parents and care providers can be trained in implementing these behavioral interventions. However, the type of training provided matters as one group of researchers found that “training” (which included direct instruction, modeling, rehearsal, roleplay and feedback on behavioral intervention) was more effective than parent “education” which included instruction on general issues regarding their children’s diagnosis and treatment options [4]. It is important to note that this study determined effectiveness using indirect measures (Aberrant Behavior Checklist—Community, ABC-C [5]; Clinical Global Impressions, CGI [6]) versus direct measures of challenging behavior. Research has also demonstrated that the effectiveness of parent-implemented behavioral interventions to reduce irritability and increase compliance was greater when parents receive the training face to face (versus blended and online) [7].

Although there is ample evidence to support the effectiveness of behavioral interventions to address challenging behavior in those with neurodevelopmental disorders, there is still disproportionate access to behavioral services across the country despite the growth in the number of Board-Certified Behavior Analysts (BCBAs) [8]. This lack of services has an impact on parents and other care providers. In a survey of 60 Quebec families with children diagnosed with intellectual and developmental disabilities (IDD) without autism spectrum disorder (ASD), researchers found that parents report a broad array of challenging behavior by their children and these parents use various strategies both as their child is engaging in behavior and prior to the occurrence of behavior in order to prevent challenging behavior [9]. Most parents felt that they were unsuccessful in their attempts to reduce their children’s challenging behavior which in turn impacted parental overall wellbeing and mental health.

One reason to focus on care provider success in managing their children’s challenging behavior is the demonstrated association between these behaviors and psychotropic medication use. In a recently published retrospective review of psychotropic medication use in children seen through a behavioral clinic over a ten-year period, researchers observed high use of psychotropic medication and polypharmacy (that is more than one psychotropic medication used to treat the same condition) [10]. This pattern persists throughout development with the presence of challenging behavior identified as a correlation for increased use of psychotropic medication in adults diagnosed with IDD [11]. Currently, however, there is limited data examining how effective behavioral interventions influence future psychotropic medication use.

Researchers have found that when children begin psychotropic medication at a young age (less than 3 years of age), they are less likely to receive behavioral intervention to address their challenging behavior [12]. Although the reported prevalence of psychotropic medication use in children within the United States has appeared to stabilize over the recent past (8.55% in 2004 to 9% in 2014) [13], the prevalence of use in children with ASD is reportedly greater than that. Shurtz and colleagues [14] examined what proportion of school-aged children with ASD were taking psychotropic medication and found that 42.5% of children in their sample were taking psychotropics with nearly half taking more than one psychotropic. Within the ASD population, behavioral and conduct disorders are largely associated with psychotropic medication use [15]. There is also a high prevalence of psychotropic medication to address challenging behavior in those with ID. In a large sample of at-risk youth (*N* = 1333), McLaren and colleagues [16] found that a majority of their sample were taking psychotropic medication (86%) with many experiencing polypharmacy (55%). Polypharmacy use warrants attention as it may have implications in the evaluation of intervention effects.

Although the effectiveness of psychotropic medication in addressing challenging behavior has been evaluated, there are limitations of the existing research (i.e., methodological considerations, small sample sizes, lack of representativeness) [17]. And, with the exception of antipsychotics, there are few clinical trial evaluations of other psychotropics. Research examining the impact of antipsychotics has largely focused on risperidone and aripiprazole, both second-generation antipsychotics, which were found to be generally effective in reducing irritability in ASD but only in children (relative to adults) [18] and both medications have side effects associated with their use, primarily weight gain, increased appetite, and sedation. Regarding antidepressants, selective-serotonin reuptake-inhibitors (or SSRIs) have been prescribed to address various behavioral concerns (e.g., repetitive behavior, aggression, and irritability) [19] with research suggesting these medications are perhaps more effective at addressing repetitive behavior rather than other challenging behavior (such as aggression and SIB) [20]. Limited research has demonstrated that serotonin-norepinephrine reuptake inhibitors (SNRIs), specifically venlafaxine or Effexor, were found to have a positive effect on SIB but at doses less than what is prescribed to treat depression [21]. The literature examining the impact of mood stabilizers and lithium on challenging behavior in neurodevelopmental disorders varies with a general lack of findings regarding the positive effect of these medications on challenging behavior (or irritability) and mild to moderate side effects were reported across the trials conducted [22]. Although psychotropic medication alone is not an effective treatment for challenging behavior, research has found that combined psychotropic medication, specifically antipsychotics, can decrease the amount of time it takes for behavioral intervention to reduce challenging behavior [23].

Given that parents are partners in the implementation of behavior intervention plans, research has examined the effectiveness of combination treatment (parent-implemented behavioral interventions and psychotropic medication). In a randomized trial comparing parent training plus risperidone to risperidone alone, Aman and colleagues [24] found that the combination treatment was more effective at reducing severe challenging behavior than medication alone as evaluated on indirect measures such as CGI and ABC-C. Although these combined treatments appear to reduce challenging behavior, it is unclear if it was the interaction of treatment modalities or if one treatment was contributing more than the other in reducing challenging behavior [25]. Research has demonstrated the effectiveness of behavior interventions when implemented by parents and the effectiveness of these interventions when used in combination with psychotropic medication. It is unclear; however, if behavioral interventions influence future psychotropic medication use. The purpose of this retrospective record review was to examine patterns of psychotropic medication use following outpatient behavioral assessment and treatment in children with neurodevelopmental disorders.

## 2. Materials and Methods

### 2.1. Participants

There were 25 children seen in our clinic who were included in this record review (21 males, 4 females). These children were between the ages of two and 16 years of age. During the first visit, the children’s average age was 4.92 years (range, 2–13). At the six-month time point, their average age was 5.48 years (range, 3–13). At the final time point, the mean age was 7.56 years (range, 4–16). All the children had comorbid diagnoses as noted in their medical files (see Table 1). Other conditions included oppositional defiance disorder, sensory difficulties, conduct disorder, and reactive attachment disorder. Topography of challenging behavior and the percentage of children who engaged in each topography is also noted in Table 1. All but two children engaged in multiple types of behavior (these two solely engaged in aggression). The “other” category of topography of challenging behavior included chewing on clothing, inappropriate topics of conversation, yelling, verbal aggression, vomiting, pica, body contortion, and dropping to the ground.

### 2.2. Behavior Clinic Description

Data presented are for children who were initially seen in an outpatient pro bono behavioral clinic over a period of two and half years. The purpose of the clinic was to conduct behavioral assessments, evaluate treatment recommendations, and train caregivers on those treatments. Given this was a pro bono clinic with a high volume of patients, follow-up visits were reserved for individuals who continued to have difficulty after their initial visit. None of the children in this review were seen for follow-up visits as the parents did not indicate a need for additional services. Prior to arriving at the clinic, care providers were contacted via telephone to conduct functional assessment interviews and to ask questions about preferred stimuli/potential reinforcers. Indirect assessments of behavior function (specifically, the Questions About Behavior Function [26] and Motivation Assessment Scale [27]) were also completed and assessments on behavior severity were captured using the ABC-C. Both assessment and treatment were evaluated in a single visit. These visits lasted approximately four hours. One patient was seen each week as the clinic was held one day a week.

During clinic visits, all assessment and treatment sessions were conducted in an exam room with a two-way mirror. The two-way mirror allowed clinicians to observe the assessment and treatment sessions and to collect data avoiding potential reactivity by the child. This room was equipped with a table, three chairs, an examination bed, and a couch. When possible, care providers were coached to conduct the functional analysis and treatment conditions (care providers delivered the intervention). Social validity questionnaires focusing on the program’s helpfulness in reducing challenging behavior, the appropriateness of recommendations, and compliance with recommendations were administered over the telephone between six weeks to three months following appointments with 20 of the care providers who attended the clinic.

### 2.3. Behavior Assessment and Interventions

Of the 25 children who were assessed, 20 received both assessment and intervention. There were two children for whom behavior was not observed during the assessment and an additional three for whom we were unable to evaluate treatment conditions. Individualized, brief functional analyses [28] were conducted to determine challenging behavior function. During these brief functional analyses, test conditions were designed to mimic evocative events (i.e., the presence of establishing operations) that were reported to lead to the presence of challenging behavior. Contingent on the occurrence of challenging behavior, caregivers delivered corresponding consequences. Test conditions were compared to control conditions. Control conditions were analog to an enriched environment with the absence of established operations present in test conditions. Based on the results of the brief functional analyses, interventions were designed to match the outcome of the test conditions. If it was determined that challenging behavior occurred to access social positive reinforcement, interventions were designed to teach the child how to engage in functionally equivalent skills to receive or maintain access to social positive reinforcement.

Caregivers were trained in how to implement the intervention procedures with their child utilizing instructions, modeling, rehearsal, and feedback. At the end of the appointment, caregivers also received written instructions on the designed intervention via a behavior intervention plan. Of the 20 children, 76% experienced a reduction in challenging behavior from assessment to treatment and half of the sample (52%) experienced a decrease in behavior greater than 80%. Reduction in behavior was determined by subtracting the mean rate of behavior observed during treatment sessions from the mean rate of behavior observed during assessment sessions. We then divided this amount by the mean rate of behavior observed in assessment sessions and multiplied by 100. We were unable to confirm whether care providers implemented interventions once they left the clinic as services were limited to the clinic on the assigned appointment day.

### 2.4. Chart Reviews

Medication records were reviewed to obtain information regarding the child’s medication status at the time of the behavioral assessment, six months following the assessment, and a later follow-up time point (i.e., a fixed date). The follow-up time point on average was 25.52 months from the six-month time period (range, 1 month to 41 months). The behavior clinic was independent of the prescriber and thus decisions made regarding psychotropic medication use were not informed by assessment or treatment delivered through the clinic. Psychotropic medications were classified by type stimulants, nonstimulant attention deficit hyperactivity disorder (ADHD) medications, antipsychotics, antidepressant SSRI, antidepressant serotonin receptor antagonists and reuptake inhibitors (SARI; which was primarily trazodone), and others. The “other” category included Namenda and hydroxyzine. There were eight categories of medication use which were grouped according to the type of medication change experienced. “Medication to No Medication” indicated that a child went from being on medication to not being on medication. “No Medication to No Medication” meant a child was not on medication at the time of the assessment and they remained medication-free. “No Medication to Medication” reflected that a child was previously not taking medication and then was taking medication. “Switch of Medications” indicated that a child had one medication discontinued and another medication started. “Same Medication” meant the medication regimen (dose and kind) remained the same. “Different Medication Added” meant that a new medication was added to the regimen. “More (Same) Medication” indicated a higher dose of the same medication. “Less (Same) Medication” indicated a lower dose of the same medication. Individuals could experience more than one type of medication change. For instance, there was one individual who went from three medications to two medications (one new and one the same). This change would be characterized as “Medication to No Medication”, “Switch of Medication”, and “Same Medication”.

## 3. Results

Table 2 presents the general changes observed with regard to the average number of medications prescribed per child and the types of medication taken at each time point. Across each time point, the average number of medications taken by each child increased. Additionally, the type of medication prescribed shifted over time. At the initial time point, more nonstimulant and stimulant medication was prescribed which was consistent with the number of children diagnosed with ADHD (32% of the sample). However, we also observed that the number of antipsychotics prescribed increased by the final time point as did the total use of antidepressants. The limited sample size and the different combinations of psychotropic medications did not allow for statistical analyses to determine if these changes were significant.

**Figure 1:**
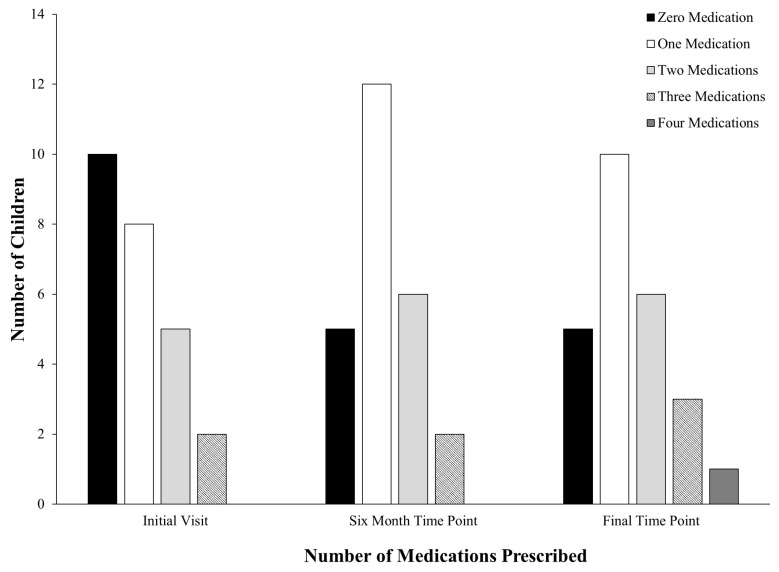
Number of psychotropic medications taken by each participant.

At the initial time point, there were more participants taking at least one psychotropic medication than not taking any psychotropics. That said, the time point in which most children were observed to not be taking psychotropic medication was in the initial time period (see Figure 1). At the six-month time point, a majority of participants were taking psychotropic medication with 12 children prescribed one psychotropic medication and five not taking any psychotropic medication. At the final time point, five children were still not taking psychotropic medication, and one child was prescribed four psychotropic medications. When examining psychotropic medication use at the final time point and treatment results in the clinic for children who experienced an 80% (*N* = 13) reduction in behavior from assessment to intervention in the clinic, we saw 61% (*N* = 8) of those children did not experience any changes in psychotropic medication regimen and 38% (*N* = 5) experienced an increase in the number of psychotropic medications. For those children who experienced less than 80% (*N* = 7) reduction in challenging behavior from assessment to intervention, 29% (*N* = 2) did not experience any changes in psychotropic medication regimen and 57% (*N* = 4) experienced an increase in the number of psychotropic medications.

**Figure 2:**
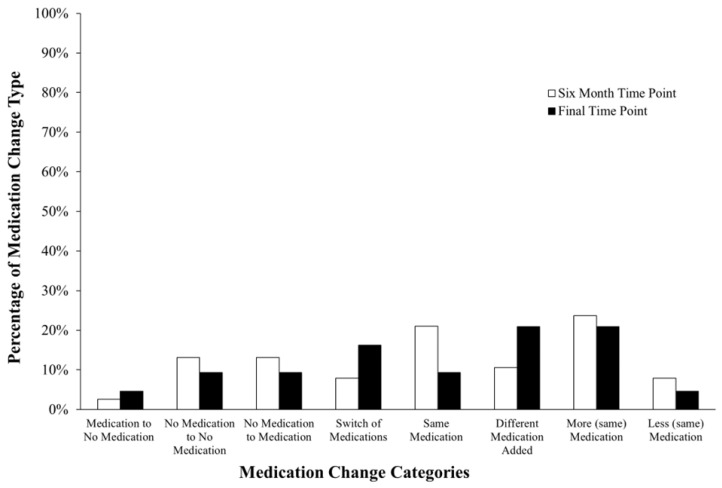
Percentage of the types of psychotropic medication changes at each time point.

This retrospective analysis also revealed that children were more likely to experience similar changes in the six months following their initial visit at the clinic when compared to the final time point (see Figure 2). The percentage of the types of psychotropic medication changes experienced at each time point was calculated by dividing the number of medication changes in each category by the total number of psychotropic medication changes observed. Most of the children in the sample experienced increases in the dose of psychotropic medication. At the six-month time point, 24% (*N* = 9) of medications prescribed had been increased, and at the final time point, 21% (*N* = 9) of the total medications prescribed had been increased in dose. There were more total types of categorical changes in psychotropic medication regimens at the final time point (*N* = 41) than at the six-month time point (*N* = 38) so the denominator was different between the two time points. Also, at the six-month time point, there was only one noted discontinuation of psychotropic medication (5%) and few observed switches of medications (8%, *N* = 3). This was similar to the final time point, with one observed discontinuation (2%) and few instances of decreases in the dosages of psychotropic medication (5%, *N* = 3). The type of medication change experienced was often different across children and time points. Many of the changes were individualized with very few participants remaining on the same medication and dose at either time point. Thus, we observed instability within each child through medication and dose.

**Figure 3:**
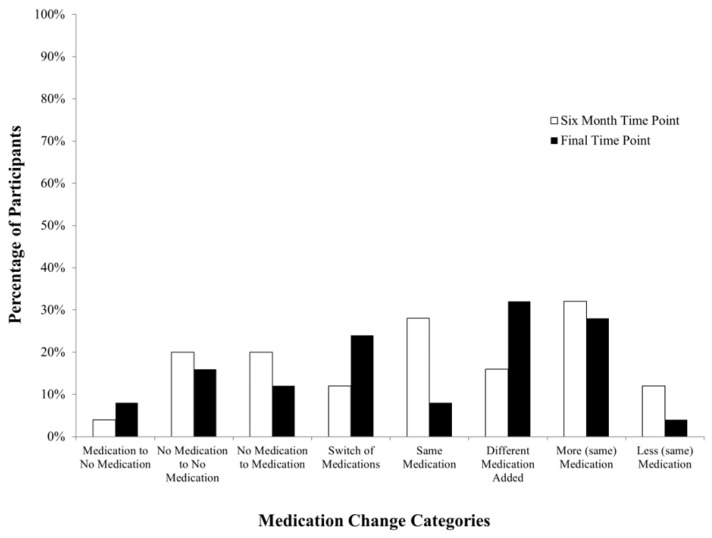
Percentage of participants experiencing each type of psychotropic medication change at each time point.

When examining the percentage of participants who experienced each type of medication change, data revealed that psychotropic medication use at the six-month time point does not necessarily correspond with future psychotropic medication use (see Figure 3). At the six-month time point, the percentage of participants who were still not using psychotropic medication (20%, *N* = 5) was the same as the percentage of participants who had new medications added to their psychotropic medication regimens (20%, *N* = 5). Additionally, a majority of participants experienced “More (same) Medication” at the six-month time point (32%, *N* = 8). Many participants also experienced no change in medication regimen (“Same Medication”; 28%, *N* = 7) at the six-month time point. Finally, at the six-month time point, only one participant had experienced a discontinuation of medication (4%) and 12% (*N* = 3) were on less of the same medication. When looking at these categories at the final time point, 16% (*N* = 4) of participants remained medication-free and 32% (*N* = 8) of participants had a new medication added to their psychotropic medication regimen. Although the same number of participants experienced “More (same) Medication” (28%, *N* = 7) at the final time point as at the six-month time point, fewer participants remained on the same medication regimen (8%, *N* = 2). And, whereas at the final time point, 8% (*N* = 2) of participants experienced discontinuation of a medication, there was only one participant who was on less of the same medication (4%).

**Figure 4:**
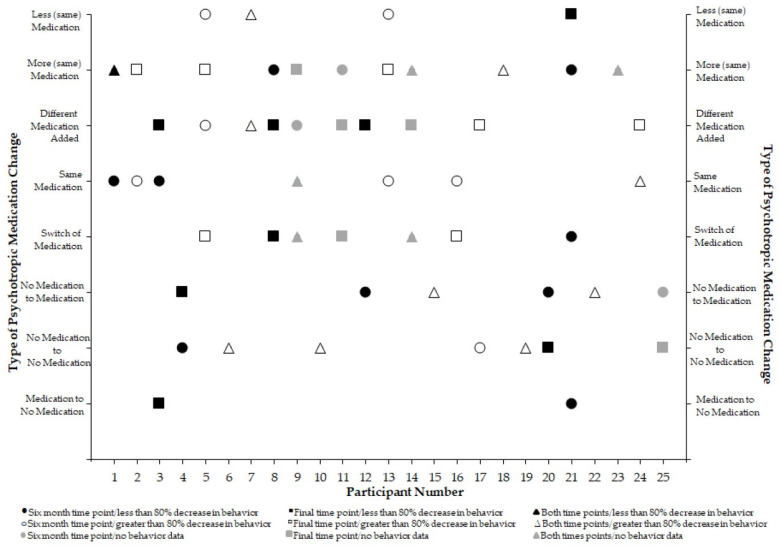
Types of medication changes experienced by each participant at each time point.

When examining changes experienced by each participant in the study, there were no consistent changes identified according to effects observed in challenging behavior during the clinical assessment and treatment (see Figure 4). That is, the reduction in challenging behavior of greater than 80% (as indicated by open symbols) or less than 80% (as indicated by closed symbols) did not appear to coincide with any specific changes in the psychotropic medication regimen. The same appears to be true for participants who did not exhibit challenging behavior during the clinic assessment or who did not receive treatment in clinic (gray symbols). Furthermore, there did not appear to be consistent changes in the medication regimen experienced by children. Changes in psychotropic medication that occurred by the 6-month time point are represented by circles, changes at the final time point are depicted with squares, and changes that were observed at both time points are represented by triangles. There was one participant (24) who at 6 months medications did not change and another participant (11) for whom two medications increased in dose. We had not observed challenging behavior in the clinic during participant 24’s assessment and had seen a greater than 80% decrease in challenging behavior for participant 11. For the final time point, there was one participant (4) who went from no medications to two medications prescribed and treatment in the clinic was not associated with decreases in behavior greater than 80%. Another participant (9) had two medications discontinued and two new medications prescribed (challenging behavior had not been observed during the assessment). Two participants (9 and 24) remained on two of the same medications. Interestingly, participant 9 did not engage in challenging behavior in the clinic and participant 24 experienced a reduction in behavior greater than 80%. Another participant (7) had two new medications added and had a greater than 80% reduction in challenging behavior during the clinic visit. Finally, there were two participants (1 and 13) who experienced increases in dose of two different psychotropics. Participant 1 did not experience a decrease of 80% in challenging behavior during treatment whereas participant 13 had. Of the participants who were assessed in the clinic, challenging behavior for 12 participants was maintained by social negative reinforcement (i.e., escape from demands). For 16 participants, challenging behavior appeared to be maintained by social positive reinforcement (e.g., access to attention or preferred items). Finally, for two participants, challenging behavior appeared to be automatically reinforced (i.e., engagement in the behavior was the reinforcer for behavior). As a reminder, there were two participants for which behavior was not observed during the assessment. These numbers are greater than 25 as often challenging behavior appeared to be maintained by more than one reinforcer. There were no discernable patterns with regard to behavior function and medication change experienced.

**Figure 5:**
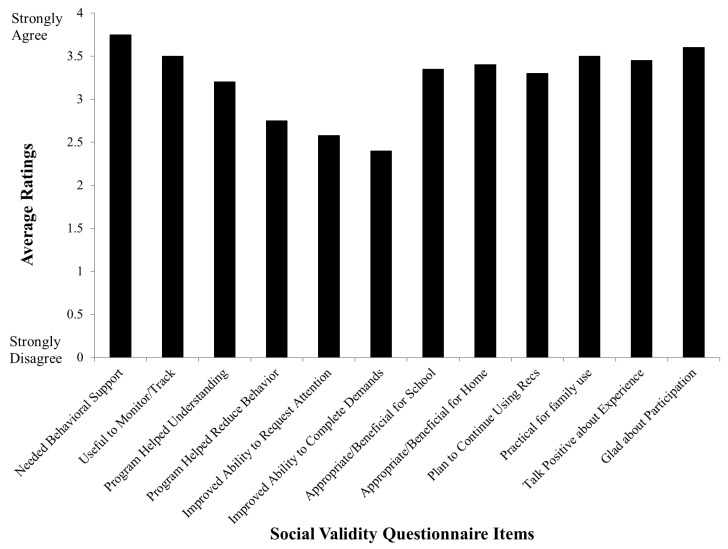
Mean scores for each item of the questionnaire evaluating social validity.

Finally, of the 25 children for whom we reviewed charts, we had 20 care providers who completed the social validity questionnaire (although we had aimed to have the questionnaires completed within six weeks to three months of the initial visit, there were some individuals with whom we did not connect). Average ratings for each item on the questionnaire were calculated (see Figure 5). The highest-rated category was a care provider feeling their child “needed behavioral support” (3.75 average) and the lowest-rated item was that their child “improved in their ability to complete demands” (2.4 average). None of the items received a zero rating by respondents. Generally, parents rated their experiences favorably with the recommendations being practical for family use (mean rating of 3.5) and being glad about their participation in the clinic (mean rating of 3.6) as being more highly rated items. We also did not discern any pattern in responses on the social validity questionnaire associated with how soon, after the clinic appointments, the questionnaires were completed.

## 4. Discussion

Previous research [29] has contended that once an individual begins psychotropic medication use, it is often difficult to discontinue psychotropic medication use, and our data are consistent with those findings. With each time point, we saw a greater average number of psychotropic medications (polypharmacy) prescribed per child and an increase in the number of children prescribed psychotropic medication. And, at the last time point, we were less likely to observe decreases in medication dose or discontinuation of psychotropic medication. One predictor of antipsychotic use is the presence of challenging behavior [30]. The children who were prescribed antipsychotics in our sample either engaged in aggression, SIB, or both behaviors.

Although the total number of children included in this review limits our ability to discuss the significance, we did observe that if challenging behavior was reduced by at least 80% during the clinic visit, those children experienced fewer changes in their medication regimen compared to those children who did not experience decreases in behavior; however, those children still experienced additions to medication regimens. This was surprising given that none of the families in this sample requested additional visits to the clinic to address their child’s behavior. Over time, the number of medications prescribed to the sample increased per child which is consistent with what has been reported in the literature [31]. For the five children who were either not fully assessed or treated due to the absence of challenging behavior during the clinic visit or running out of time during the visit, four of the children were observed to experience increases in the dose and additions of medication whereas one child remained medication free. Finally, although care providers generally rated their experiences with the clinic as favorable, additions and increases to psychotropic medication regimens still occurred. Thus, ratings on our measures of social validity of the clinic experience did not appear to correspond with less future psychotropic medication use.

Admittedly, there are limitations to this work. The behavioral assessments and treatments delivered through this clinic were brief and time limited. Although the fidelity of treatment implementation was assessed in vivo with feedback delivered immediately we do not have treatment fidelity data to report. Further, we did not collect follow-up data on challenging behavior. Based on this, it is impossible to determine if the behavioral interventions developed and trained through the clinic impacted future psychotropic medication use for these children. An additional limitation is that there was no way to assess the fidelity of adherence to treatment recommendations. That is, we were unable to determine if parents followed the recommendations provided within the behavioral intervention plan despite parental reporting their intention to do so. The only follow-up completed was with the social validity questionnaire but questions regarding adherence to the recommendations were not explicitly posed. We also did not assess behavior severity or topography at the follow-up time points to determine if medication changes were related to the behavior targeted at the clinic or if other challenging behavior emerged. For example, stimulant and nonstimulant medication were common classes of psychotropics used and manipulated. It is possible that there were other classes of behavior (e.g., inattention or hyperactivity) that were driving psychotropic medication changes that were not targeted during the behavioral assessments and treatments. Finally, the varying lengths of time between the six-month and final time points across the participants make it difficult to determine patterns. However, those children with final time points occurring in less than a year from the six-month time point were found to experience increases and additions to their psychotropic medication regimen similar to those who had greater than a year between time points. Ultimately, the data presented here are limited to the current sample and cannot be generalized beyond this sample given the small sample size.

Future research should determine if effective behavioral interventions when adhered to and implemented with high fidelity by care providers impact future psychotropic medication use in children. Looking at when behavioral services are more robust (i.e., delivered continuously or extended intervention delivery) and by whom (i.e., either therapist or caregiver implemented) may also be an avenue to explore. Given how prevalent psychotropic medication use within this population is and the known adverse side effects associated with psychotropic medication, expanding access to treatments that reduce the likelihood of medication use is vital. Research should also explore if the social validity of interventions, as rated by care providers, predicts adherence to behavioral plans and what potential impact social validity has on future psychotropic medication use. Finally, it would be worthwhile to explore if the function of challenging behavior is associated with psychotropic medication use. For example, is behavior maintained by negative reinforcement more likely to result in psychotropic medication use than behavior maintained by access to positive reinforcement?

## 5. Conclusions

Children with neurodevelopmental disorders are more likely to be prescribed psychotropic medication than their neurotypical peers [32]. Our research suggests that when children with neurodevelopmental disorders access brief behavioral assessment and intervention to address challenging behavior, psychotropic medications are often a component of treatment; however, we observed that greater reductions in behavior were associated with fewer medication changes for this sample of children. What drives the continued use of psychotropic medication even when effective behavioral interventions are identified is unclear. There is limited evaluation of care provider’s social validity or acceptability of psychotropic medication use by their children [33]. Often, behavioral interventions require changes in environments, that is that caregivers change their method of interaction with children and potentially rearrange stimuli within those environments (i.e., home, school). Care providers often have unrealistic expectations of the impact of psychotropic medication on behavior [33] and one can see how these expectations may develop as preliminary research suggests that decreases observed in challenging behavior coinciding with medication changes may just be coincidental [34]. Clinicians working with families should consider an educational approach to behavioral and pharmacological treatment whereby realistic expectations for the time, effort, magnitude, and stability of behavior change are thoroughly discussed. This approach requires prescribers and behavior interventionists to work together to evaluate treatment response. Future research should examine different models of integrated care to identify early factors that predict psychotropic medication use and evaluate whether comprehensive early intervention, which facilitates the development of adaptive skills and addresses challenging behavior as it develops, impacts the likelihood children are prescribed psychotropic medication. Prevention is important as this study, consistent with others, found that once a child begins psychotropic medication, they are not likely to stop using psychotropic medication and they are more likely to experience polypharmacy.

## Figures and Tables

**Table 1:** Participant diagnoses and topography of challenging behavior.

Diagnoses	Percentage of Children
Receptive-Expressive Language delay	40%
Autism Spectrum disorder	36%
Attention-Deficit Hyperactivity disorder	32%
Intellectual disability	24%
Genetic/Chromosomal disorder	16%
Anxiety disorder	8%
Seizure disorder	8%
Other conditions	36%
Topography of Challenging Behavior	
Aggression	88%
Noncompliance	40%
Property Destruction/Disruption	40%
Self-injury	24%
Tantrums	24%
Elopement	12%
Other	36%

**Table 2:** Psychotropic medication use observed at each time point.

	Initial Assessment	6 Month Follow Up	Final Follow Up
Average number of medications used	0.96 medications (range, 0–3)	1.2 medications (range, 0–3)	1.4 medications (range, 0–4)
Type (*N*) of Medication Prescribed	Stimulants—7 Nonstimulants—11 Antipsychotics—5 Antidepressants SSRI—1 Antidepressants SARI—0 Other—0	Stimulants—8 Nonstimulants—14 Antipsychotics—5 Antidepressants SSRI—0 Antidepressants SNRI—2 Other—1	Stimulants—9 Nonstimulants—12 Antipsychotics—9 Antidepressants SSRI—2 Antidepressants SNRI—3 Other—1

## Data Availability

Data are unavailable due to privacy restrictions.

## Abbreviations

The following abbreviations are used in this manuscript:

IDD	Intellectual and Developmental Disabilities
ASD	Autism Spectrum Disorder
SIB	Self-injurious Behavior
ID	Intellectual Disability
ABC-C	Aberrant Behavior Checklist-Community
CGI	Clinical Global Impressions
